# Premature Labor Induced by Quinine

**Published:** 1881-04

**Authors:** 


Premature Labor Induced by Quinine.—It is sometimes questioned whether quinine does or does not induce labor when given to pregnant women. It is not often necessary to give it in England; but in India, where ague is so common, it often becomes a serious question whether to give it or withhold it in pregnancy. A case has just occurred to me which bears out the idea that quinine does induce labor.

A lady placed herself in my hands; she expected her confinement the latter part of August. In the middle of July she had bad fever, which originally of an intermittent kind, showed a tendency to become continuous. I endeavored, with some success at first, to keep off the fever with jaborandi and arseniate of quinine, without giving the sulphate of quinine. The amount of quinine in the arseniate was so small that I did not fear giving it. Nearly a month passed, and the fever was so bad and intractable, and she became so weak, that I began to fear the consequences to both mother and child; so, on the 7th of August, I resolved to give quinine. I gave it in five-grain doses, which I ordered three times a day. She took two doses—one in the morning and the second at mid-day. At about 8 o'clock in the evening, without any warning, the "waters burst," and slight uterine pains came on. They were, however, very slight—so much so that she doubted whether she was in labor at all, until at about 11 o'clock the pains became a little stronger; one or two sharp pains succeeded, and the child's head, followed after a few moments by the body, was born. The placenta came away about half an hour afterwards without hæmorrhage. The child was very small, and looked as though it had been born a month before its time. A great deal of its skin peeled off, and after a week it became very much jaundiced, but it promises to do well.

There can be little doubt that the moderate doses of quinine which I gave caused the birth of the child, and under the circumstances it was perhaps the best thing that could have happened. But I shall be careful that I do not give quinine to a pregnant woman again unless I feel that I am obliged to do so. In the case which I have recorded I deliberately chose the induction of labor as a less dangerous alternative than the continuance of the fever.—*London Lancet.*

